# Development and Validation of an Individualized Metabolism-Related Prognostic Model for Adult Acute Myeloid Leukemia Patients

**DOI:** 10.3389/fonc.2022.829007

**Published:** 2022-06-17

**Authors:** Cong Wei, Lijuan Ding, Qian Luo, Xiaoqing Li, Xiangjun Zeng, Delin Kong, Xiaohong Yu, Jingjing Feng, Yishan Ye, Limengmeng Wang, He Huang

**Affiliations:** ^1^ Bone Marrow Transplantation Center, The First Affiliated Hospital, Zhejiang University School of Medicine, Hangzhou, China; ^2^ Liangzhu Laboratory, Zhejiang University Medical Center, Hangzhou, China; ^3^ Institute of Hematology, Zhejiang University, Hangzhou, China; ^4^ Zhejiang Province Engineering Laboratory for Stem Cell and Immunity Therapy, Hangzhou, China

**Keywords:** acute myeloid leukemia, MRPSI, MCPMI, prognosis, drug response

## Abstract

**Objectives:**

Acute myeloid leukemia (AML) is a highly heterogeneous hematologic malignancy with widely variable prognosis. For this reason, a more tailored-stratified approach for prognosis is urgently needed to improve the treatment success rates of AML patients.

**Methods:**

In the investigation of metabolic pattern in AML patients, we developed a metabolism-related prognostic model, which was consisted of metabolism-related gene pairs (MRGPs) identified by pairwise comparison. Furthermore, we analyzed the predictive ability and clinical significance of the prognostic model.

**Results:**

Given the significant differences in metabolic pathways between AML patients and healthy donors, we proposed a metabolism-related prognostic signature index (MRPSI) consisting of three MRGPs, which were remarkedly related with the overall survival of AML patients in the training set. The association of MRPSI with prognosis was also validated in two other independent cohorts, suggesting that high MRPSI score can identify patients with poor prognosis. The MRPSI and age were confirmed to be independent prognostic factors *via* multivariate Cox regression analysis. Furthermore, we combined MRPSI with age and constructed a composite metabolism-clinical prognostic model index (MCPMI), which demonstrated better prognostic accuracy in all cohorts. Stratification analysis and multivariate Cox regression analysis revealed that the MCPMI was an independent prognostic factor. By estimating the sensitivity of anti-cancer drugs in different AML patients, we selected five drugs that were more sensitive to patients in MCPMI-high group than those in MCPMI-low group.

**Conclusion:**

Our study provided an individualized metabolism-related prognostic model that identified high-risk patients and revealed new potential therapeutic drugs for AML patients with poor prognosis.

## Introduction

Acute myeloid leukemia (AML) is the term for a family with high heterogeneous hematological malignancies, which are caused by diverse phenotypic and genetic alterations that affect the differentiation of hematopoietic stem and progenitor cells (HSPCs). These abnormalities impede HSPCs differentiation at various stages and lead to clonal expansion of myeloid blasts in the bone marrow, peripheral blood, and other tissues (1). Despite current treatments involving intensive chemotherapy, targeted therapies and hematopoietic stem cell transplantation (HSCT), AML remains lethal for approximately 50% of young patients and 80% of elder patients due to relapse, primary resistance, or treatment-related mortality (2, 3). The diagnosis and prognostic stratification of AML are mainly based on age, cytogenetic characteristics, molecular subtypes and other histological markers (4). Accurate risk stratification of AML patients is necessary for precise identification of high-risk patients who may benefit from advanced treatment (2, 5). Thus, additional factors that can be used for prognostic stratification are urgently needed to improve the outcomes of AML patients.

Tumor cells share a common phenotype of unrestrained proliferation and consume extremely large amounts of energy and metabolites (6, 7). In recent years, many studies have shown that tumor cells metabolism differs significantly from normal cells, and the difference can be exploited to diagnose, monitor, and treat cancer patients (8–10). Metabolomic studies have revealed that different types of tumor cells have distinct metabolic phenotypes that vary according to genetic alterations, epigenetic features and gene dependencies (11). Glucose metabolism, which is closely related to therapeutic resistance and clinical outcomes, has been confirmed to be altered in many cancers (12, 13). Evaluation of serum metabolomic differences between AML patients and healthy controls demonstrated alterations in multiple metabolic pathways, including biosynthesis of proteins and lipoproteins, glycolysis, the tricarboxylic acid (TCA) cycle, and metabolism of choline and fatty acids (14, 15). For example, both cholesterol synthesis and low-density lipoprotein (LDL) processing are hyperactive in AML cells, and cholesterol levels rise dramatically following sublethal doses of radiation or chemotherapeutics (16, 17). In addition, certain gene mutations in AML also cause metabolic changes. 10% - 20% AML patients harbor mutations in isocitrate dehydrogenases (IDHs) (18), critical enzymes in the TCA cycle, and mutant IDH1/2 with a neomorphic activity that converts alpha-ketoglutarate (α-KG) to 2-hydroxyglutarate (19, 20). Mutations of the FMS-like tyrosine kinase-3 (FLT3) are found in approximately 30% of newly diagnosed AML patients (21, 22). FLT3 is a transmembrane ligand-activated receptor tyrosine kinase that regulates cell survival, proliferation, and differentiation through various signaling pathways (23, 24). To improve the prognosis of AML patients, efforts should be made to discover novel and sensitive metabolic markers that could be used to recognize patients with poor prognosis and optimize treatment strategies. Based on the above reports, we speculated that genes involved in metabolism could serve as prognosis-related gene signatures and be used to predict the long-term survival of AML patients.

In this study, we explored 318 differentially expressed metabolism-related genes (MRGs) between AML patients and healthy donors by analyzing the gene expression data from The Cancer Genome Atlas (TCGA) and Genotype-Tissue Expression (GTEx) databases. To eliminate technical bias caused by data from different platforms, the metabolism-related prognostic signature index (MRPSI) was generated from three MRG pairs (MRGPs) with prognostic values, which were derived from the relative ranking of MRGs. The predictive performance of the MRPSI was evaluated by determining the area under the curve (AUC) value of the receiver operating characteristic (ROC) curve. And the results of Kaplan-Meier survival analysis showed that the MRPSI-low patients had significantly better survival in comparison with the MRPSI-high patients. Next, the MRPSI was further validated using the GSE12417 and GSE37642 databases. The MRPSI and age were confirmed to be independent prognostic factors *via* multivariate Cox regression analysis. To fully exploit the predictive potential of the clinical and molecular characteristics of AML patients, we integrated MRPSI with age, yielding a metabolic-clinical prognostic model (MCPM), which allowed us to estimate AML prognosis with improved accuracy. Stratification analysis and multivariate Cox regression analysis revealed that the MCPMI was an independent prognostic factor for AML patients. Furthermore, we estimated half-maximal inhibitory concentration (IC50) values for clinical drugs and found that AML patients with a high MCPM index (MCPMI) exhibited enhanced sensitivity to five of these drugs. The results of these analyses provide important new information shedding light on the metabolic profile of AML and can be used to improve the accuracy of risk stratification and better predict the survival of patients.

## Materials and Methods

### Sample Collection and Study Design

As displayed in the analysis pipeline (**Supplementary Figure S1**), data were collected from 730 AML patients and 70 healthy donors (151 AML patients from the TCGA cohort and 70 healthy donors from the GTEx cohort were used to develop the signature, 162 AML patients from the GSE12417 cohort were used to test the signature, and 417 AML patients from the GSE37642 cohort were used to validate the signature). And all data included in this study were obtained before treatment.

Fragments per kilobase of exon model per million mapped fragments (FPKM) data were downloaded from bone marrow samples of 151 AML patients included in the TCGA database (https://portal.gdc.cancer.gov/) and 70 healthy donors included in the GTEx database (https://www.gtexportal.org/home/). First, we identified 2338 genes that showed differential expression between normal and abnormal bone marrow using the “limma” package with the following criteria: false discovery rate (FDR) < 0.05 and log2(fold change) > 1. Next, among 2031 MRGs from the publicly accessible ccmGDB database (http://bioinfo.mc.vanderbilt.edu/ccmGDB) (25), 318 MRGs that differentially expressed in TCGA cohort were identified. The microarray datasets for the GSE12417 and GSE37642 cohorts were obtained from the Gene Expression Omnibus (GEO, http://www.ncbi.nlm.nih.gov/geo) under accession numbers GSE12417 and GSE37642 (26, 27). The characteristics and clinical outcomes of the three cohorts are listed in **Supplementary Table S1**.

### Generation of a Prognostic Model Using MRGs

Out of 318 MRGs acquired from the TCGA cohort, 67 genes were shared in all datasets. Therefore, we used the 67 shared MRGs to generate 505 MRGPs. A pairwise comparison was performed between the metabolism-related gene expression value in each sample to obtain a score of 0 or 1 for each MRGP. When the expression value of the first gene in an MRGP was greater than the second gene, the MRGP score was 1, otherwise MRGP score was 0 (28). The chosen method was entirely dependent on the gene expression profile of each individual tumor sample and did not require normalization. Next, by utilizing univariate Cox proportional hazards regression modeling, we determined the prognostic value of 505 MRGPs using the TCGA dataset. Then we applied LASSO Cox proportional hazards regression modeling (glmnet R software) to reduce the potential for overfitting, and we selected the minimum criteria (29). To optimize the prognostic signature and allow it to be applied more easily, multivariate Cox proportional hazards regression was used to select a set of MRGPs, to form the MRPS that was used to make prediction. The MRPSI was calculated using a formula in which the scores of the selected MRGPs were weighted according to their coefficients. Next, we selected the optimal cut-off value for classifying the subjects of the study into MRPSI-high and MRPSI-low groups using a time-dependent ROC curve (survival ROC, version 1.0.3) at five years in the training cohort (28). The predictive power of the MRPSI for OS was investigated *via* Kaplan-Meier survival and ROC analyses using three independent cohorts. In addition, we conducted univariate and multivariate Cox regression analyses to determine whether MRPSI was an independent prognostic risk factor.

### Development and Validation of MCPM

The MRPSI and age were integrated into the MCPM using a LASSO Cox proportional hazards regression model in the TCGA dataset based on the results of the multivariate Cox regression analyses in the three cohorts mentioned above. The MCPMI was calculated for each sample *via* a linear combination of the selected parameters and weighted by the corresponding coefficients. Similar to the aforementioned method for determining the optimal cutoff of the MRPSI, the optimal cutoff value for the MCPMI was also determined on the basis of the ROC analysis. ROC and Kaplan-Meier survival analyses were applied to assess the prognostic performance of the MCPMI in three cohorts. In addition, we conducted univariate and multivariate Cox regression analyses to investigate the independent prognostic performance of the MCPMI.

### Clinical Drug Response Prediction

First, a list of commonly used clinical drugs was acquired from the Genomics of Drug Sensitivity in Cancer (GDSC) (https://www.cancerrxgene.org/) database, and prediction analysis was conducted using the R package “pRRophetic”. The IC50 of each sample was estimated using ridge regression. All parameters were set to their default values, and mean values were used to identify duplicate gene expression (30, 31).

### Statistical Analysis

All statistical analyses in this study were conducted using R software (version 3.6.0) and GraphPad Prism software (version 5.0). Kaplan-Meier survival analysis with the log-rank test was conducted to compare survival curves for subgroup analysis. Statistical analyses between pairs of groups were conducted using the chi-square and Mann-Whitney U tests. All P-values are reported in this study as two-tailed values. For all results, the threshold for significance was P-value < 0.05, unless otherwise specified.

## Results

### Comparison of the Metabolic Phenotypes of Bone Marrow Samples From AML Patients and Healthy Donors

**Figure 1:**
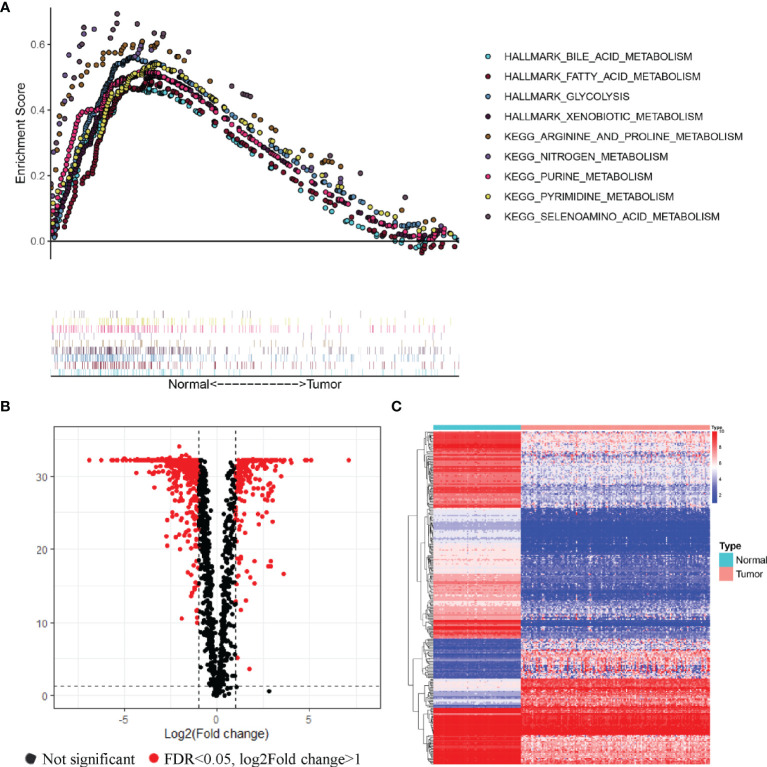
Relationships of the metabolic phenotype of the bone marrow samples between AML patients and healthy donors. **(A)** GSEA analysis of gene expression profiles in AML and normal bone marrow. Significant enrichment of metabolic pathways was found in the bone marrow samples of healthy donors, compared with AML patients. **(B, C)** Volcano plot **(B)** and heatmap **(C)** of metabolism-related genes that were differentially expressed in AML samples compared to healthy donors.

We obtained gene expression data from the bone marrow samples of 151 AML patients and 70 healthy donors from the TCGA and GTEx databases. The distinct features of biological processes associated with AML were investigated by performing gene set enrichment analysis (GSEA) using normalized mRNA expression data. The GSEA results showed that the gene expression data of healthy donors were significantly enriched in diverse metabolic pathways in comparison with the AML patients (**Figure 1A**). Enriched pathways in healthy donors related to metabolic functions included metabolism of bile acid (NES = 1.88, P < 0.001), metabolism of fatty acid (NES = 1.75, P < 0.001), glycolysis (NES = 1.83, P < 0.001), xenobiotic metabolism (NES = 1.78, P < 0.001), metabolism of arginine and proline (NES = 2.05, P < 0.001), metabolism of nitrogen (NES = 1.92, P < 0.001), metabolism of purine (NES = 2.11, P < 0.001), metabolism of pyrimidine (NES = 2.06, P < 0.001), and metabolism of selenoamino acid (NES = 1.99, P < 0.001). To further explore the differences between the metabolic phenotypes of AML patients and healthy donors, an analysis of expression profiles of 2,031 metabolism-related genes obtained from the ccmGDB database was performed. Among the analyzed genes, 318 MRGs were found to be differentially expressed in AML patients compared with healthy donors (**Figures 1B, C**).

### Construction and Definition of the MRPSI for AML in the TCGA Set

**Figure 2:**
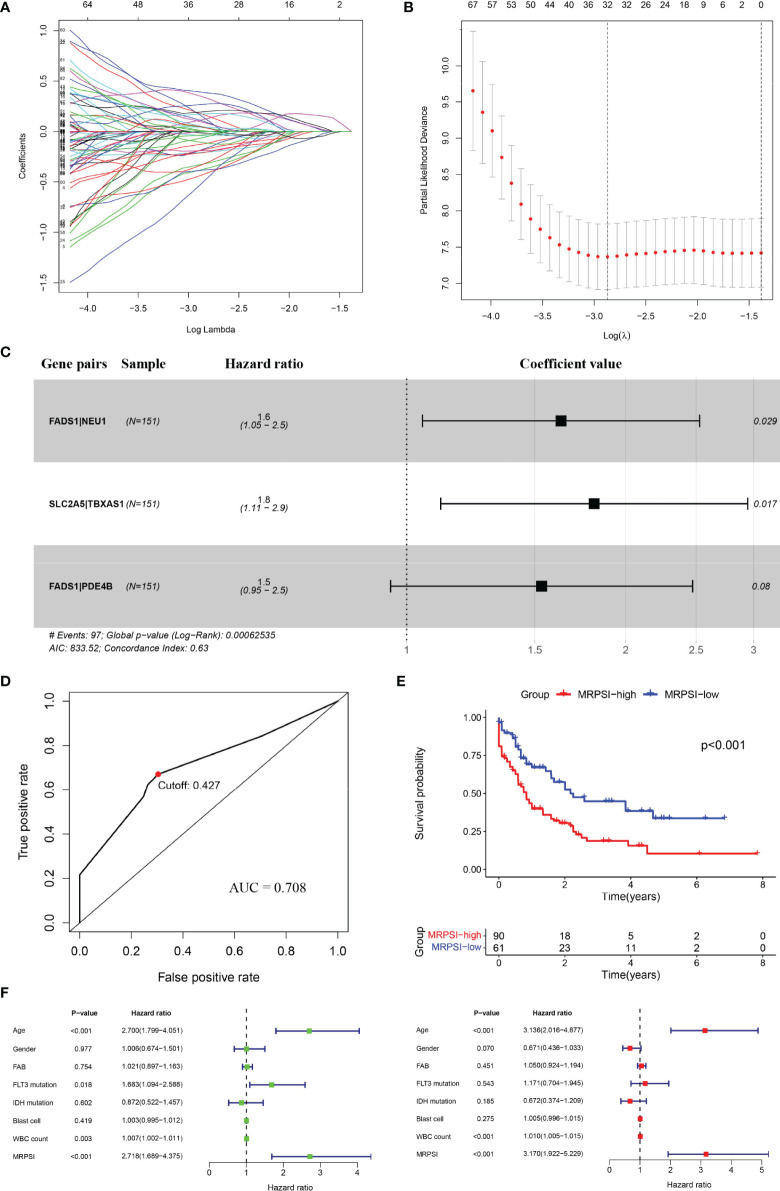
Construction and definition of the MRPSI for AML in the TCGA set. **(A, B7)** Screening diagram of Lamda **(A)** and regression coefficient **(B)** in the LASSO Cox regression analysis. **(C)** The three selected gene pairs included in the signature, their hazard ratios and coefficient values using multivariate Cox regression analysis. **(D)** ROC curve of overall survival for AML patients using the MRPSI in the TCGA cohort. **(E)** Kaplan-Meier curves for overall survival analysis of AML patients using the MRPSI in the TCGA cohort. **(F)** Univariate (left) and multivariate (right) Cox regression analyses of the MRPSI and clinical factors for the predictive value of overall survival in the TCGA cohort.

Given the significant differences in metabolic reprogramming between AML patients and healthy donors revealed in the above analysis, we next generated a prognostic signature based on MRGs. We found that 67 out of the 318 MRGs acquired from the TCGA dataset were included in all datasets, and pairwise comparison was used to generate 505 MRGPs. Univariate Cox proportional hazards regression modeling was utilized to select 122 prognostic MRGPs that had significant relationships with OS (P < 0.05). Next, we applied LASSO Cox proportional hazards regression analysis and selected 32 MRGPs with the optimal predictive performance from the set of 122 prognostic MRGPs according to criteria described above (**Figures 2A, B**). Afterwards, with the optimized and practical values of the prognostic signatures taken into consideration, we applied multivariate Cox regression analysis to construct a novel predictive MRPS, which consisted of three gene pairs (**Figure 2C**). **Supplementary Table S2** showed the three selected MRGPs and their corresponding coefficients. Finally, we calculated the MRPSI score of each patient according to the following formula: MRPSI score = 0.489 × value of FADS1| NEU1 +0.594 × value of SLC2A5| TBXAS1 +0.427 × value of FADS1| PDE4B. Using the optimal cutoff value of 0.427, the patients were classified as the MRPSI-high group (n=90) or the MRPSI-low group (n=61).

To determine the predictive utility of the MRPSI, we calculated the AUC value of the ROC, subsequently we performed Kaplan-Meier survival analysis. The AUC value of the MRPSI for five-year overall survival (OS) was 0.708 (**Figure 2D**). In comparison with the MRPSI-low group, the patients in the MRPSI-high group had significantly worse OS (P < 0.001; **Figure 2E**). Clinical factors including age, gender, French-American-Britain (FAB) classification, FLT3 mutation, IDH mutation, blast cell, white blood cell (WBC) count and MRPSI were taken into account in univariate and multivariate Cox regression analyses of the training set. We found that MRPSI, age and WBC count were independent prognostic factors (MRPSI: P < 0.001; age: P < 0.001; WBC count: P < 0.001; **Figure 2F**). Considering that hazard ratio of age was far greater than 1, ROC and Kaplan-Meier survival analysis were also performed to determine the prognostic performance of age. The Kaplan-Meier survival analysis showed significant difference in the OS of patients younger than 60 years old (n=88) and that of patients older than 60 years old (n=63) (P < 0.001; **Supplementary Figure S2B**). However, the AUC value of MRPSI was greater than that of age (age: AUC = 0.657; **Supplementary Figure S2A**). We also analyzed the prognostic performance of MRPSI in patients with different age status and WBC count. The results of Kaplan-Meier survival analysis in the TCGA cohort showed that among subgroups, long-term survival times in MRPSI-high group were not always remarkably shorter than those of MRPSI-low group (**Supplementary Figures S3A–D**).

### Validation of the MRPSI for AML

**Figure 3:**
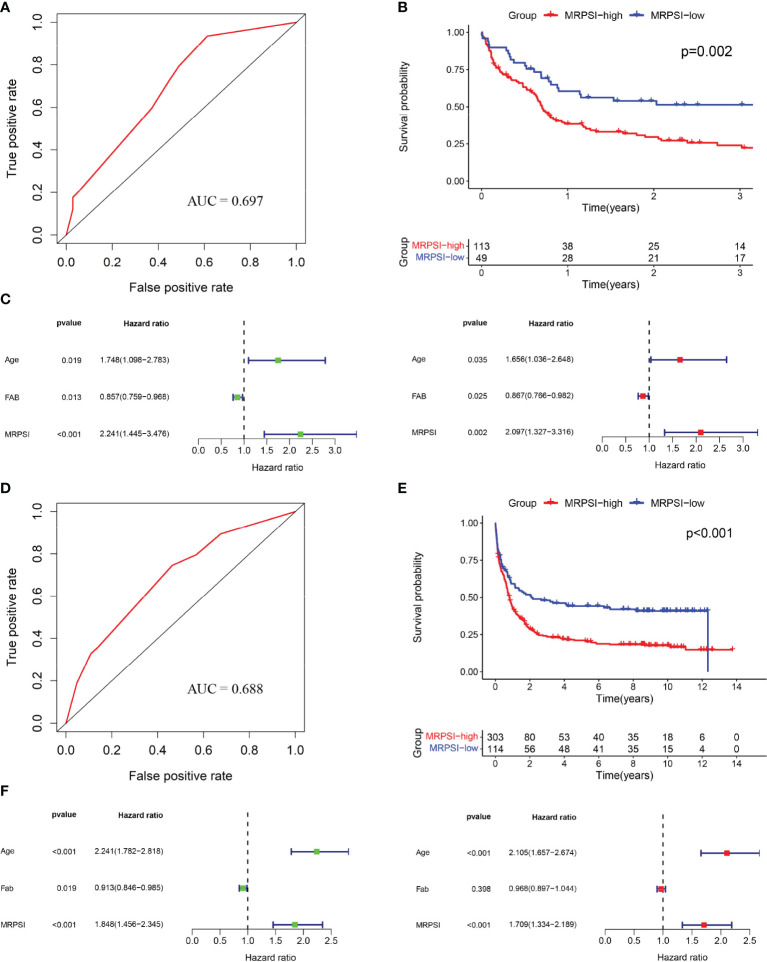
Validation of the MRPSI for AML. **(A)** ROC analysis of overall survival for the MRPSI in the GSE12417 cohort. **(B)** Kaplan-Meier curves for overall survival analysis of AML patients based on the MRPSI in the GSE12417 cohort. **(C)** Univariate (left) and multivariate (right) Cox regression analyses of the MRPSI and clinical factors for the predictive value of overall survival in the GSE12417 cohort. **(D)** ROC analysis of overall survival for the MRPSI in the GSE37642 cohort. **(E)** Kaplan-Meier curves for overall survival analysis of AML patients based on the MRPSI in the GSE37642 cohort. **(F)** Univariate (left) and multivariate (right) Cox regression analyses of the MRPSI and clinical factors for the predictive value of overall survival in the GSE37642 cohort.

To verify the discriminative ability of the novel MRPSI to group AML patients based on OS, the formula described above was applied to the test set from the GSE12417 cohort. The 162 patients in the cohort were classified as the MRPSI-low group (n=49) or MRPSI-high group (n=113) based on the cutoff value of the training set. The AUC value of the MRPSI for five-year OS for AML patients in the test set was 0.697, indicating that the MRPSI was reliable as a prognostic signature (**Figure 3A**). The results of the Kaplan-Meier survival analysis demonstrated that the OS of the patients in the MRPSI-high group was significantly worse than the patients in the MRPSI-low group (P = 0.002; **Figure 3B**). Moreover, according to the univariate and multivariate Cox regression analyses, we found that MRPSI and age were predictive factors (MRPSI: P < 0.001; age: P = 0.019; **Figure 3C**) and significant independent predictive factors of OS (MRPSI: P = 0.002; age: P = 0.035; **Figure 3C**). The Kaplan-Meier survival analysis revealed significant difference in the OS of patients younger than 60 years old (n=88) in comparison with patients older than 60 years (n=74) (P = 0.003; **Supplementary Figure S2D**). In agreement with the results described above, the AUC value of MRPSI was greater than that of age (age: AUC=0.617; **Supplementary Figure S2C**). When the prognostic performance of MRPSI in patients at different ages was analyzed, we found that the OS in MRPSI-low group was not higher than that of the MRPSI-high group in the AML patients older than 60 years old (**Supplementary Figure S3E, F**).

To confirm the reliability and utility of the MRPSI, we investigated its prognostic value using an independent cohort (GSE37642). Notably, the MRPSI was a robust prognostic signature for AML patients, with an AUC value of 0.688 for five-year OS (**Figure 3D**). Next, the 417 patients in the GSE37642 cohort were classified into MRPSI-high (n=303) and MRPSI-low (n=114) groups based on the same cut-off value used above. Kaplan-Meier survival analysis demonstrated that the long-term survival of the MRPSI-high and MRPSI-low groups differed significantly (P < 0.001; **Figure 3E**). According to the univariate and multivariate Cox regression analyses, we found that the MRPSI and age were both independent prognostic factors for OS (MRPSI: P < 0.001; age: P < 0.001; **Figure 3F**). Thus, ROC and Kaplan-Meier survival analyses were performed to determine the prognostic performance of age, which revealed that the AUC value of age for OS was 0.688 (**Supplementary Figure S2E**). In addition, Kaplan-Meier survival analysis also showed that the OS of patients younger than 60 years old (n=226) was significantly higher than that of patients older than 60 years old (n=191) (P < 0.001; **Supplementary Figure S2F**). And the Kaplan-Meier survival analysis showed that, regardless of the age subgroup, patients in MRPSI-high group had poorer OS than those in MRPSI-low group (P < 0.05; **Supplementary Figures S3G, H**). In addition, we summarized the distribution of patients in MRPSI-high and MRPSI-low groups concerning clinical factors, including age, gender, FAB classification, FLT3 mutation, IDH mutation, blast cell, and WBC count in **Supplementary Table S3**. The results revealed that FLT3 mutation and IDH mutation had no effect on the grouping of risk groups. However, the difference about the distribution of FAB classification in MRPSI-high and MRPSI-low groups was observed in the three datasets.

To identify the distinct features of biological processes between the patients in MRPSI-high and MRPSI-low groups, we performed GSEA using gene expression data from 417 AML patients in the GSE37642 cohort. The GSEA results showed that the gene expression data of patients in MRPSI-low group were significantly enriched in diverse metabolic pathways in comparison with those in the MRPSI-high group (**Supplementary Figure S4**). Enriched pathways in MRPSI-low group related to glycolysis (NES = -1.42, P = 0.04), xenobiotic metabolism (NES = -1.42, P = 0.04), and adipogenesis (NES = -1.46, P = 0.04). Notably, compared to those in the MRPSI-high group, patients in MRPSI-low group were also remarkably enriched in apoptosis pathway (NES = -1.53, P = 0.008) and complement pathway (NES = -1.48, P = 0.03).

### Development of the MCPMI by Combining the MRPSI With Age

**Figure 4:**
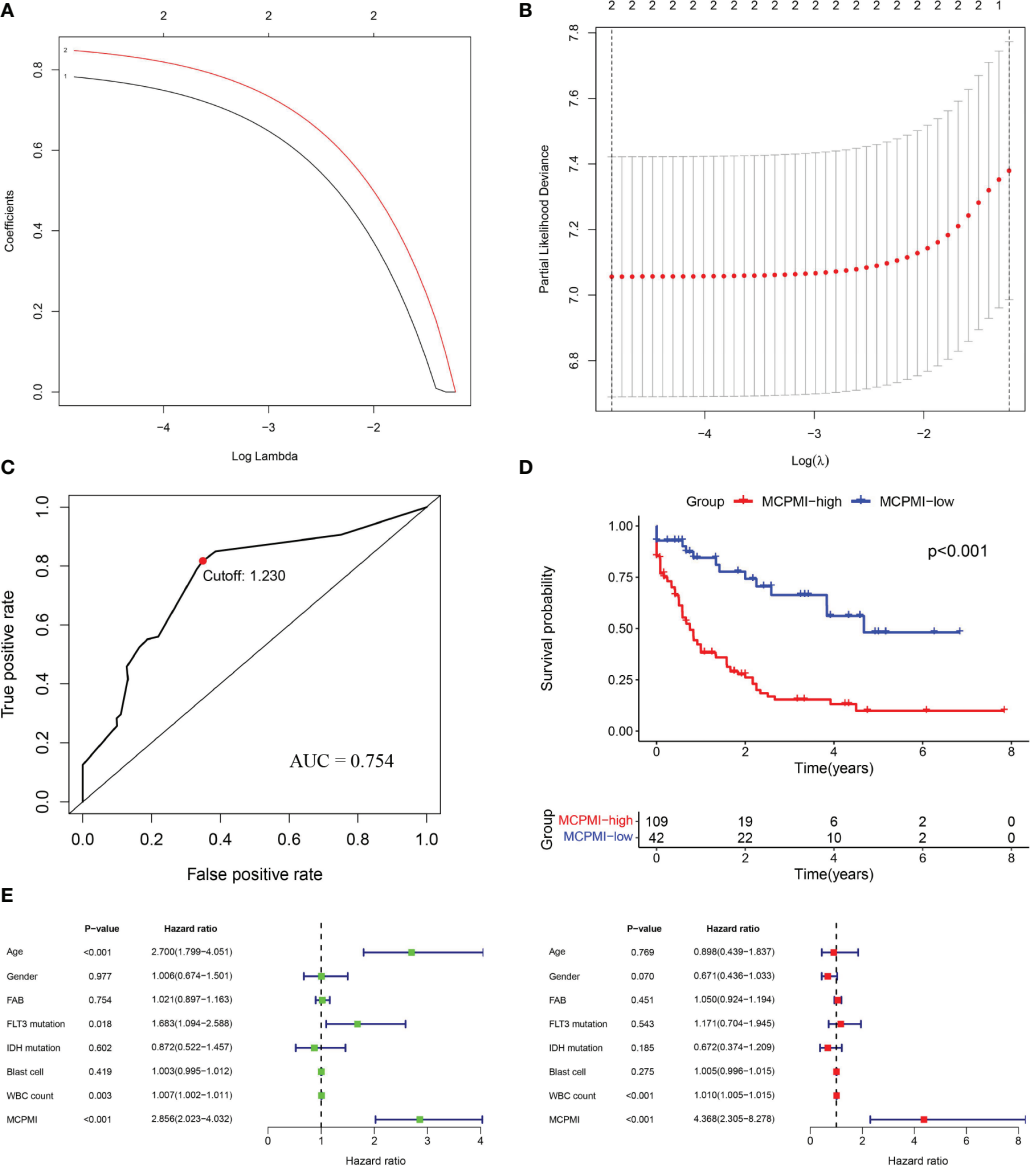
Generation of the MCPMI by combining the MRPSI and age in the TCGA cohort. (A and B) Screening diagram of Lamda **(A)** and regression coefficient **(B)** in the LASSO Cox regression analysis. **(C)** ROC curve of overall survival for AML patients using the MCPMI in the TCGA cohort. **(D)** Kaplan-Meier curves for overall survival analysis of AML patients using the MCPMI in the TCGA cohort. **(E)** Univariate (left) and multivariate (right) regression analyses of the MCPMI and clinical factors for the predictive value of overall survival in the TCGA cohort.

**Figure 5:**
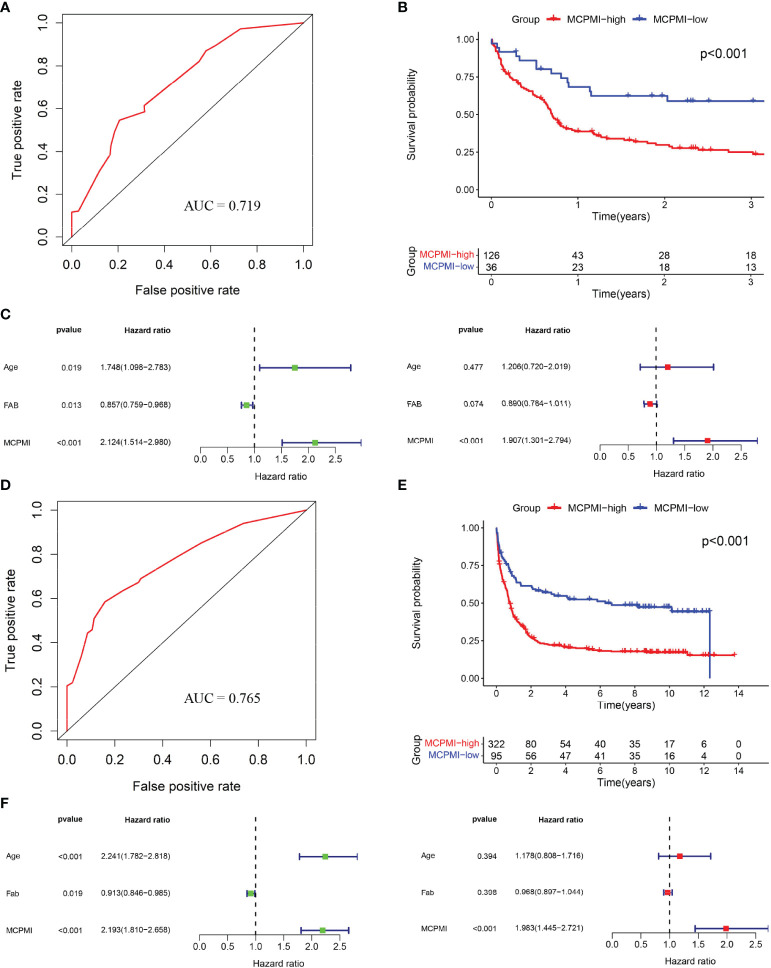
Validation of the MCPMI for AML patients. **(A)** ROC analysis of overall survival for the MCPMI in the GSE12417 cohort. **(B)** Kaplan-Meier curves for overall survival analysis of AML patients using the MCPMI in the GSE12417 cohort. **(C)** Univariate (left) and multivariate (right) Cox regression analyses of the MCPMI and clinical factors for the predictive value of overall survival in the GSE12417 cohort. **(D)** ROC analysis of overall survival for the MCPMI in the GSE37642 cohort. **(E)** Kaplan-Meier curves for overall survival analysis of AML patients using the MCPMI in the GSE37642 cohort. **(F)** Univariate (left) and multivariate (right) Cox regression analyses of the MCPMI and clinical factors for the predictive value of overall survival in the GSE37642 cohort.

Both MRPSI and age were found to be independent prognostic risk factors in multivariate Cox regression analysis of three independent datasets, suggesting that these two factors could be utilized together and complement each other to improve the prognostic value of the signature described above. Therefore, a LASSO Cox proportional hazards regression model was applied to the TCGA dataset to generate the MCPMI by combining the MRPSI score with age (**Figures 4A, B**). Subsequently, the formula of the MCPMI was determined to be: MCPMI = 0.783 × MRPSI score + 0.848 × age score. Using the ROC analysis of the TCGA set, the optimal cutoff value for assigning patients to each group was determined to be 1.230. After the patients were classified into MCPMI-high and MCPMI-low groups in three cohorts according to the formula defined above, ROC and Kaplan-Meier survival analyses were performed to assess the prognostic performance of the MCPMI. The MCPMI was confirmed to be a reliable prognostic model for AML patients. For the five-year OS of the AML patients, the AUC values of the TCGA cohort, the GSE12417 cohort and the GSE37642 cohort were 0.754, 0.719, and 0.765, respectively (**Figures 4C**, **5A, D**). Kaplan-Meier survival analysis showed that the OS of patients in the MCPMI-high group were significantly worse than that of patients in the MCPMI-low group (P < 0.001; **Figures 4D**, **5B, E**). In addition, univariate Cox regression analysis revealed that the MCPMI was a predictive factor for OS (P < 0.001; **Figures 4E**, **5C, F**). Moreover, according to multivariate Cox regression analysis, we found that the MCPMI had independent prognostic value for OS when modified for other clinical characteristics (P < 0.001; **Figures 4E**, **5C, F**). Although WBC count was also an independent prognostic factor, its hazard ratio was almost equal to 1 in TCGA dataset (P < 0.001, hazard ratio = 1.010; **Figure 4E**). Furthermore, we summarized the distribution of patients in MCPMI-high and MCPMI-low groups concerning clinical factors mentioned previously in **Supplementary Table S4**. The results showed a difference in age distribution between the MCPMI-high and MCPMI-low groups. Then we analyzed the prognostic performance of MCPMI in patients with different age status and WBC count. The results of Kaplan-Meier survival analysis in the three cohorts showed that among subgroups, long-term survival times in MRPSI-high groups were always shorter than those of MRPSI-low groups (P < 0.05; **Supplementary Figure S5**).

### Relationship Between the MCPMI and Drug Response in AML Patients

**Figure 6:**
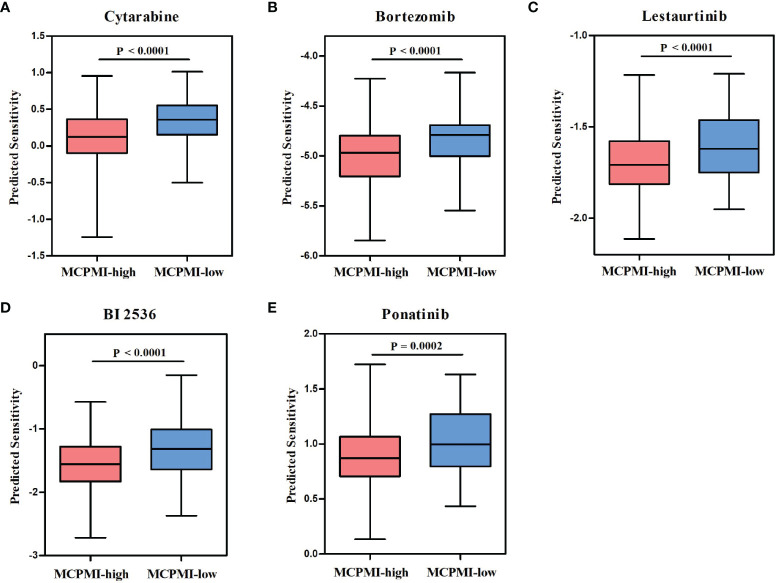
Relationships between the MCPMI and drug responses in AML patients. **(A-E)** Boxplots evaluating responses to the chemotherapeutics cytarabine **(A)**, bortezomib **(B)**, lestaurtinib **(C)**, BI 2536 **(D)** and ponatinib **(E)** between MCPMI-high and MCPMI-low patients.

Chemotherapy and targeted therapy are commonly used in the comprehensive treatment regimens for AML patients. Therefore, we used the R package “pRRophetic” to assess the sensitivity of AML patients to clinically used anti-cancer drugs based on gene expression levels. By estimating the IC50 for each sample in the GSE37642 cohort of 417 AML patients, five drugs (cytarabine, bortezomib, lestaurtinib, BI 2536, and ponatinib) were found to have significantly greater response sensitivity for MCPMI-high patients in comparison with MCPMI-low patients (cytarabine: P < 0.0001; bortezomib: P < 0.0001; lestaurtinib: P < 0.0001; BI 2536: P < 0.0001; ponatinib: P = 0.0002; **Figure 6**). In addition, MRPSI-high patients also showed greater sensitivity to these drugs in comparison with MRPSI-low patients (cytarabine: P < 0.0001; bortezomib: P < 0.0001; lestaurtinib: P < 0.0001; BI 2536: P < 0.0001; ponatinib: P = 0.0004; **Figure S6**).

## Discussion

AML is an extremely common type of acute leukemia characterized by low survival rates. Analyses of immunophenotyping, cytochemistry and cytogenetics are necessary for risk stratification and treatment guidance in AML patients (4, 32). However, the interval between diagnosis of AML and its prognosis varies greatly from patient to patient, thus requiring more precise disease stratification methods and more sensitive drug screening techniques to improve and prolong the survival of AML patients. In the last few decades, tumor metabolism has been proved to be of vital importance in tumorigenesis, tumor progression, tumor metastasis, responses to therapeutics, and prognosis. Therefore, tumor metabolism has become a focus of precision medicine research both in hematological malignancies and solid tumors (12, 32). Many metabolism-associated pathways have been shown to be extraordinarily useful for prognostic prediction for AML patients (33, 34). However, limited studies have been reported of a comprehensive metabolic signature with utility as a predictor for AML patient prognosis and survival.

In this study, we found significant enrichment of diverse metabolic pathways in the gene expression profiles of healthy donors in comparison with AML patients, including bile acid metabolism, fatty acid metabolism, glycolysis, xenobiotic metabolism, and metabolism of arginine, proline, nitrogen, purine, pyrimidine, and selenoamino acid. A comparison with metabolism-related genes from the ccmGDB database revealed that 318 MRGs were differentially expressed between AML patients and healthy donors. Subsequently, to eliminate technical bias as much as possible, we used the 67 shared MRGs in all three datasets to generate 505 MRGPs. Based on MRGPs with prognostic values, we constructed a novel formula called MRPSI to predict the long-term survival of AML patients, and then divided patients into MRPSI-high and MRPSI-low groups based on the optimal cutoff value. Kaplan-Meier survival analysis showed that the MRPSI-high patients had significantly worse OS compared with the MRPSI-low patients. Furthermore, univariate and multivariate Cox regression analyses demonstrated that the MRPSI was an independent predictive factor for the OS of AML patients. Consistent results were obtained when we applied an identical formula and cutoff value to the training, test and validation sets. Based on these strengths, the MRGP-related signature described here could be translated into clinical practice to predict the survival of AML patients and contribute to personalized patient management.

The findings of the multivariate Cox regression analysis demonstrated that age was an independent prognostic risk factor for AML patients. Recent studies showed that age at diagnosis of AML patients significantly affected survival (35, 36). In comparison with younger adults, older AML patients frequently show unfavorable cytogenetics, multidrug resistance and poor outcomes (37). Therefore, we exploited the complementary predictive values of the MRPSI and age to generate the MCPMI. Integration of the MRPSI and age improved the predictive efficacy and accuracy of survival assessments for AML patients in three independent cohorts. In comparison with the MRPSI, the novel prognostic formula of MCPMI possessed a higher AUC value. Moreover, stratification analysis and multivariate Cox regression analysis revealed that the MCPMI was an independent prognostic factor. Compared to MRPSI, the prognostic performance of MCPMI was always stable. These results indicated that the MCPMI could be an effective prognostic tool for AML patients in the future.

Improved patient stratification and the identification of new targets for anti-cancer therapeutics could be achieved using prognostic and predictive biomarkers derived from studies of the metabolic microenvironment in AML. We identified five candidate drugs for the treatment of AML from a set of frequently used clinical drugs from the GDSC database, namely, cytarabine, bortezomib, lestaurtinib, BI 2536, and ponatinib. These drugs are currently used clinically or in clinical trials to treat AML patients. Cytarabine (Ara-C), as an antimetabolic drug, interfered with cell proliferation by inhibiting the synthesis of DNA, which has been one of the most commonly used chemotherapeutic agents in the treatment of AML for a relatively long period of time (3, 38). Bortezomib is a boronic acid peptide that inhibits the 26S proteasome by binding and inhibiting the chymotrypsin-like catalytic domain of the 20S proteasome core (39, 40). Moreover, bortezomib was the first proteasome inhibitor to be approved to treat patients with multiple myeloma or mantle cell lymphoma (41–43). A study also reported bortezomib could be applied to treat metabolic disorders *via* the attenuation of the endoplasmic reticulum stress palmitic induced by palmitic acid (44). Lestaurtinib (CEP-701) is an indolocarbazole alkaloid compound that is orally bioavailable and inhibits the activity of FLT3, Janus kinase 2 (JAK2), tropomyosin receptor kinases and neurotrophin receptors (45–47). In a phase 2 clinical trial, lestaurtinib was used as a first-line treatment for AML patients of relatively advanced age (48, 49). BI 2536, a selective inhibitor of polo-like kinase 1 (PLK1) (50), has been used in several clinical trials as a treatment for patients with AML (Clinicaltrials.gov: NCT00701766) and non-small-cell lung cancer (Clinicaltrials.gov: NCT02211833). Ponatinib (AP24534) is a breakpoint cluster region-Abelson (BCR-ABL) inhibitor that was shown to be capable of overcoming mutation-based resistance in chronic myeloid leukemia patients (51, 52). Recent studies have shown that ponatinib may have clinical promise as a fibroblast growth factor receptor (FGFR) inhibitor for the treatment of particular patient populations (53). Our analysis demonstrated that MCPMI-high patients were more sensitive to cytarabine, bortezomib, lestaurtinib, BI 2536, and ponatinib than MCPMI-low patients, and these drugs may provide better treatment options for MCPMI-high patients.

The limitation of this study was mainly the retrospective analysis. However, we attempted to include as many diverse datasets as possible to increase the rigor of our signature validation process. In addition, although we successfully identified five drugs with higher sensitivity in AML patients with high MCPMI, further robust prospective studies and clinical trials are needed to assess the clinical utility of these drugs across patients with different characteristics.

In conclusion, our findings provided crucial new insight into the metabolic profile of AML patients. We provided a composite metabolism and clinical model as a novel prognostic stratification method and identified several potential therapeutic drugs for AML patients with poor prognosis.

## Data Availability Statement

The original contributions presented in the study are included in the article/**Supplementary Material**. Further inquiries can be directed to the corresponding authors.

## Author Contributions

CW and LD designed the study, performed experiments, analyzed data, and wrote the manuscript. QL, XL, XZ, DK, XY, JF, and YY performed experiments and analyzed data. HH and LW conceived and designed the study and wrote the manuscript. All authors contributed to the article and approved the submitted version.

## Funding

This work was supported by grants from the National Natural Science Foundation of China (81730008, 82000180, 82000179), Zhejiang Provincial Natural Science Foundation of China (No. LY19H080008) and the Key Project of Science and Technology Department of Zhejiang Province (2020C03G2013586).

## Conflict of Interest

The authors declare that the research was conducted in the absence of any commercial or financial relationships that could be construed as a potential conflict of interest.

## Publisher’s Note

All claims expressed in this article are solely those of the authors and do not necessarily represent those of their affiliated organizations, or those of the publisher, the editors and the reviewers. Any product that may be evaluated in this article, or claim that may be made by its manufacturer, is not guaranteed or endorsed by the publisher.
