# Supplementary material for: Development and Validation of an Individualized Metabolism-Related Prognostic Model for Adult Acute Myeloid Leukemia Patients
Source: Front Oncol. 2022 Jun 17;12:829007. doi: 10.3389/fonc.2022.829007 (PMC9247176; doi:10.3389/fonc.2022.829007)
Supplement: Supplementary file 4 [file DataSheet_1.docx]

Supplementary Material

## Supplementary Figures


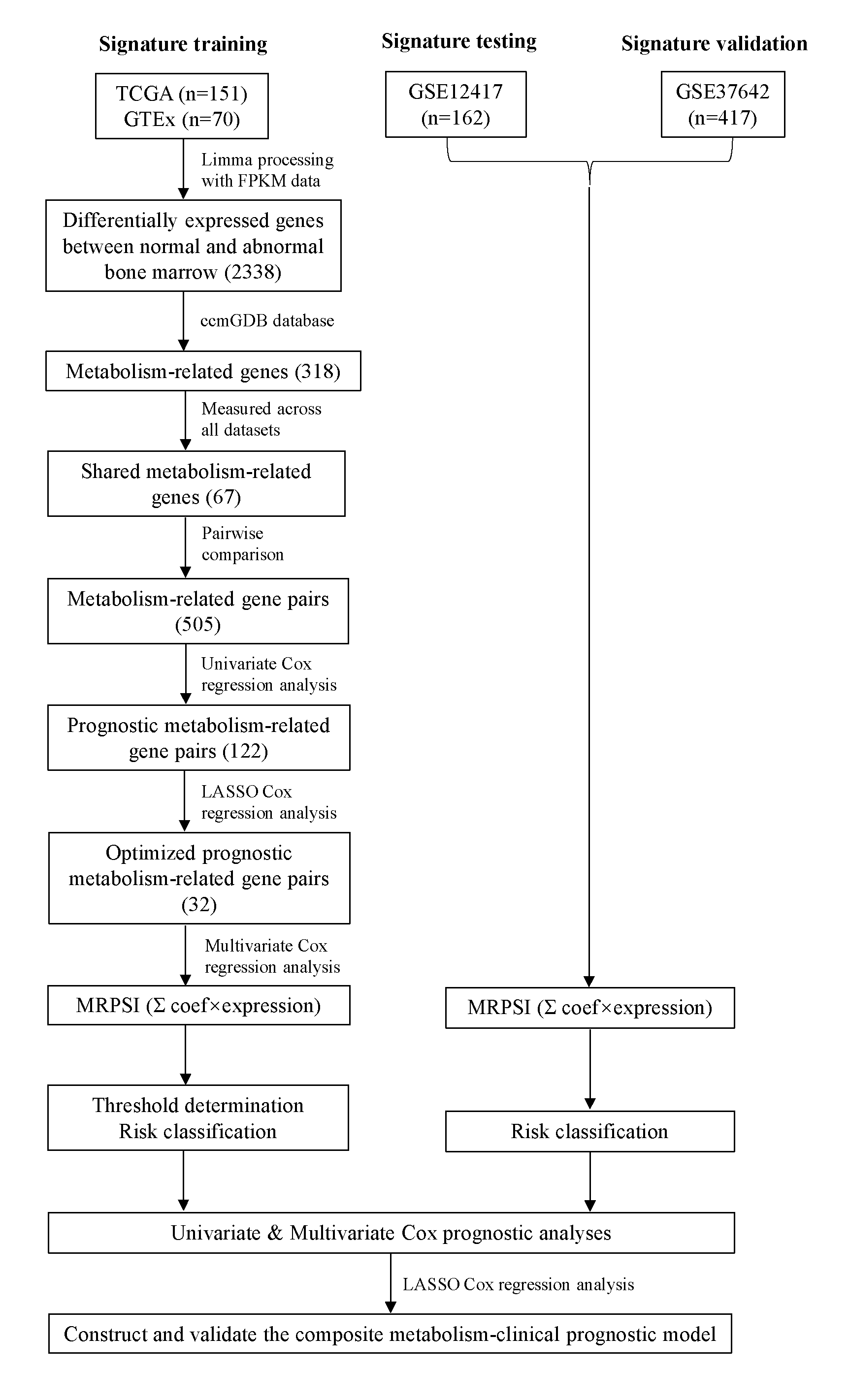


**Supplementary Figure 1.** The analysis pipeline of the construction and validation of MRPSI in AML patients.

**
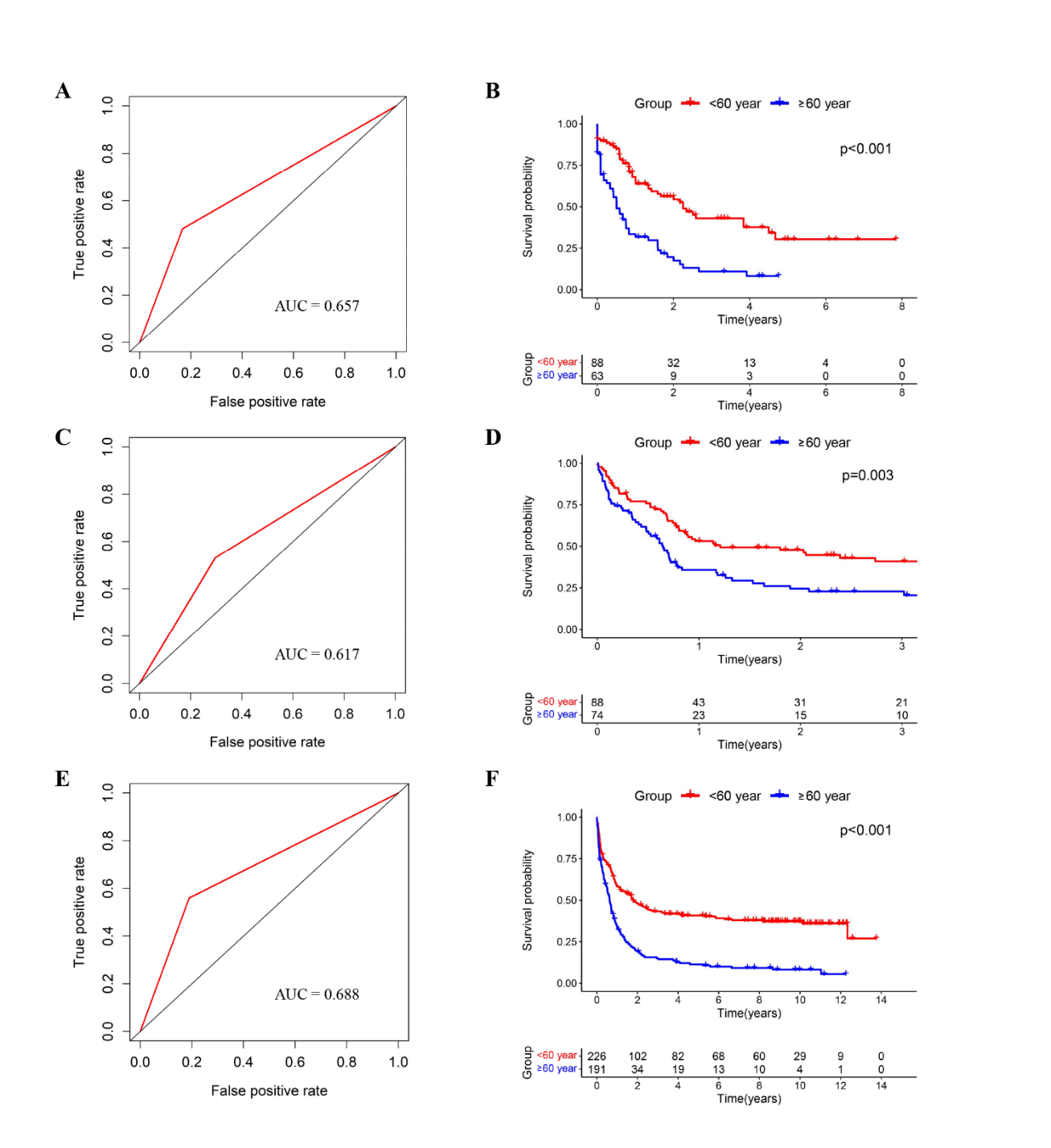
**

**Supplementary Figure 2.** Prognostic analysis of the age in AML patients. (A) ROC analysis of the age for overall survival in the TCGA set. (B) Kaplan-Meier survival curves of overall survival for AML patients based on the age in the TCGA set. (C) ROC analysis of the age for overall survival in the GSE12417 set. (D) Kaplan-Meier survival curves of overall survival for AML patients based on the age in the GSE12417 set. (E) ROC analysis of the age for overall survival in the GSE37642 set. (F) Kaplan-Meier survival curves of overall survival for AML patients based on the age in the GSE37642 set.

**
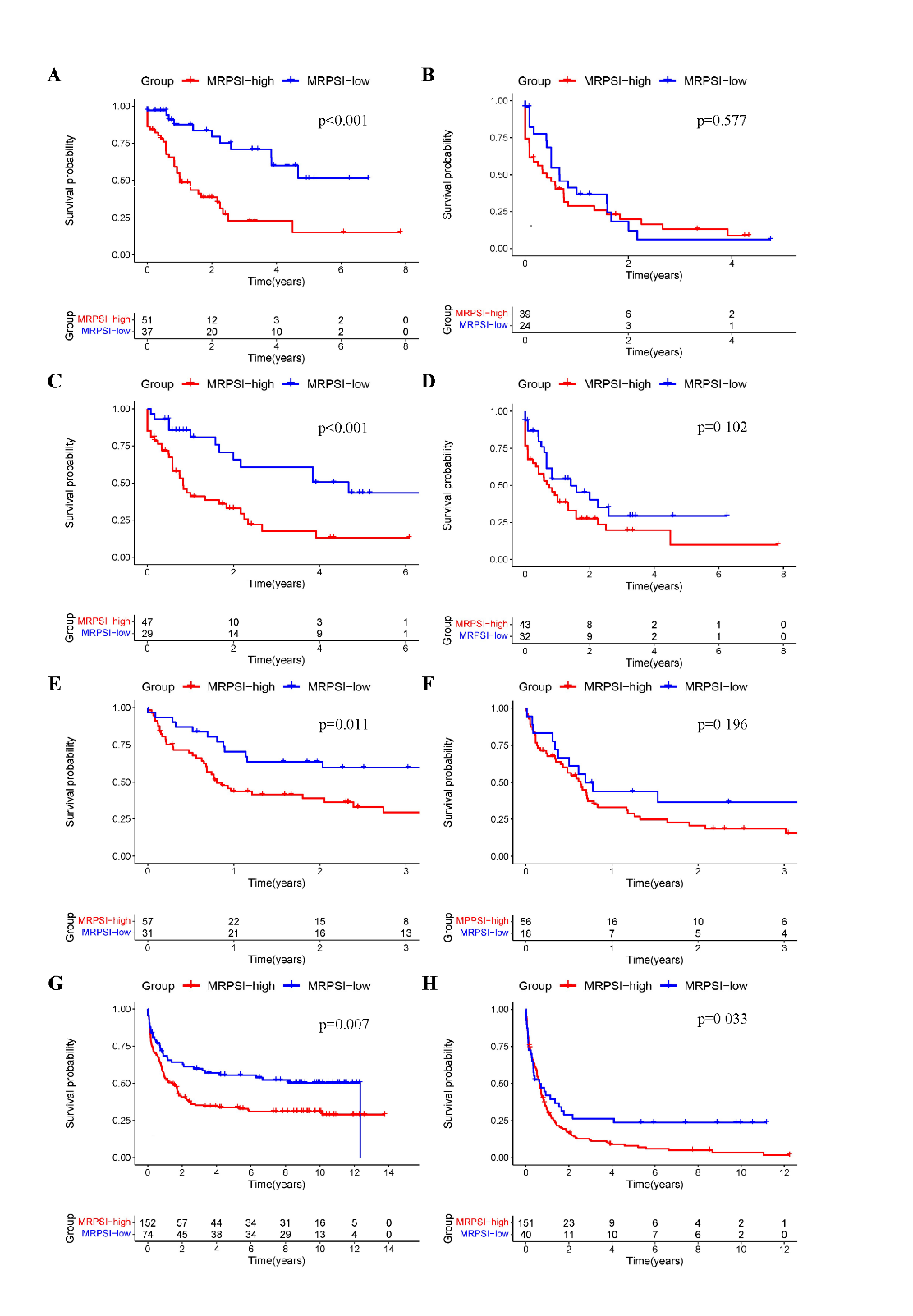
**

**Supplementary Figure 3.** Stratification analysis of MRPSI for its prognostic value. (A and B) Kaplan-Meier survival curves of overall survival for AML patients younger than 60 years (A) and older than 60 years (B) based on the MRPSI in the TCGA set. (C and D) Kaplan-Meier survival curves of overall survival for AML patients with WBC count＜19^9/L (C) and WBC count≥19^9/L (D) based on the MRPSI in the TCGA set. (E and F) Kaplan-Meier survival curves of overall survival for AML patients younger than 60 years (E) and older than 60 years (F) based on the MRPSI in the GSE12417 set. (G and H) Kaplan-Meier survival curves of overall survival for AML patients younger than 60 years (G) and older than 60 years (H) based on the MRPSI in the GSE37642 set.


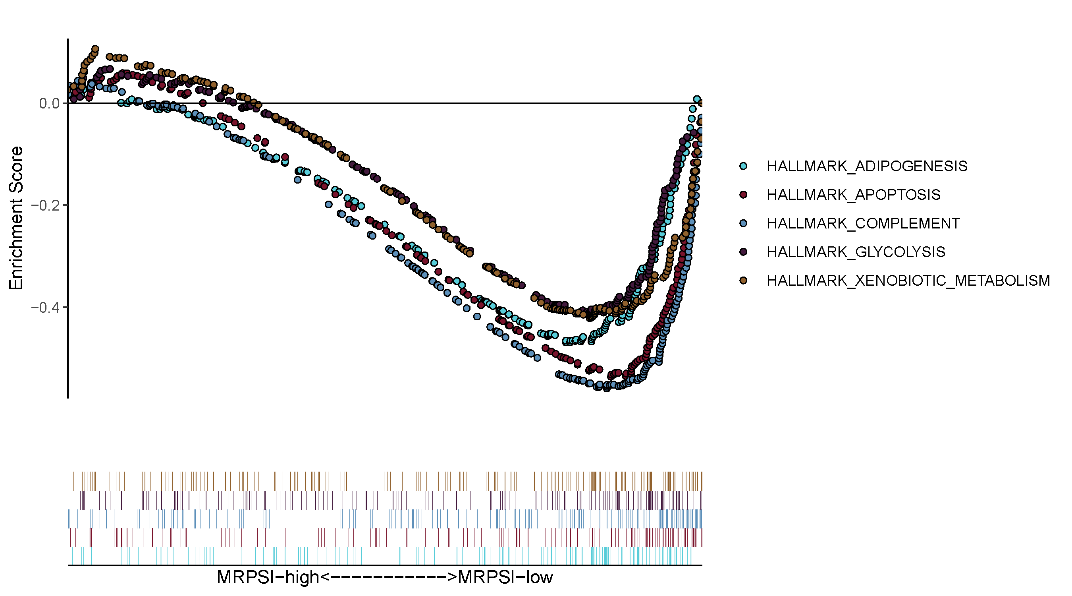


**Supplementary Figure 4.** GSEA analysis of gene expression profiles in patients of MRPSI-high and MRPSI-low groups.

**
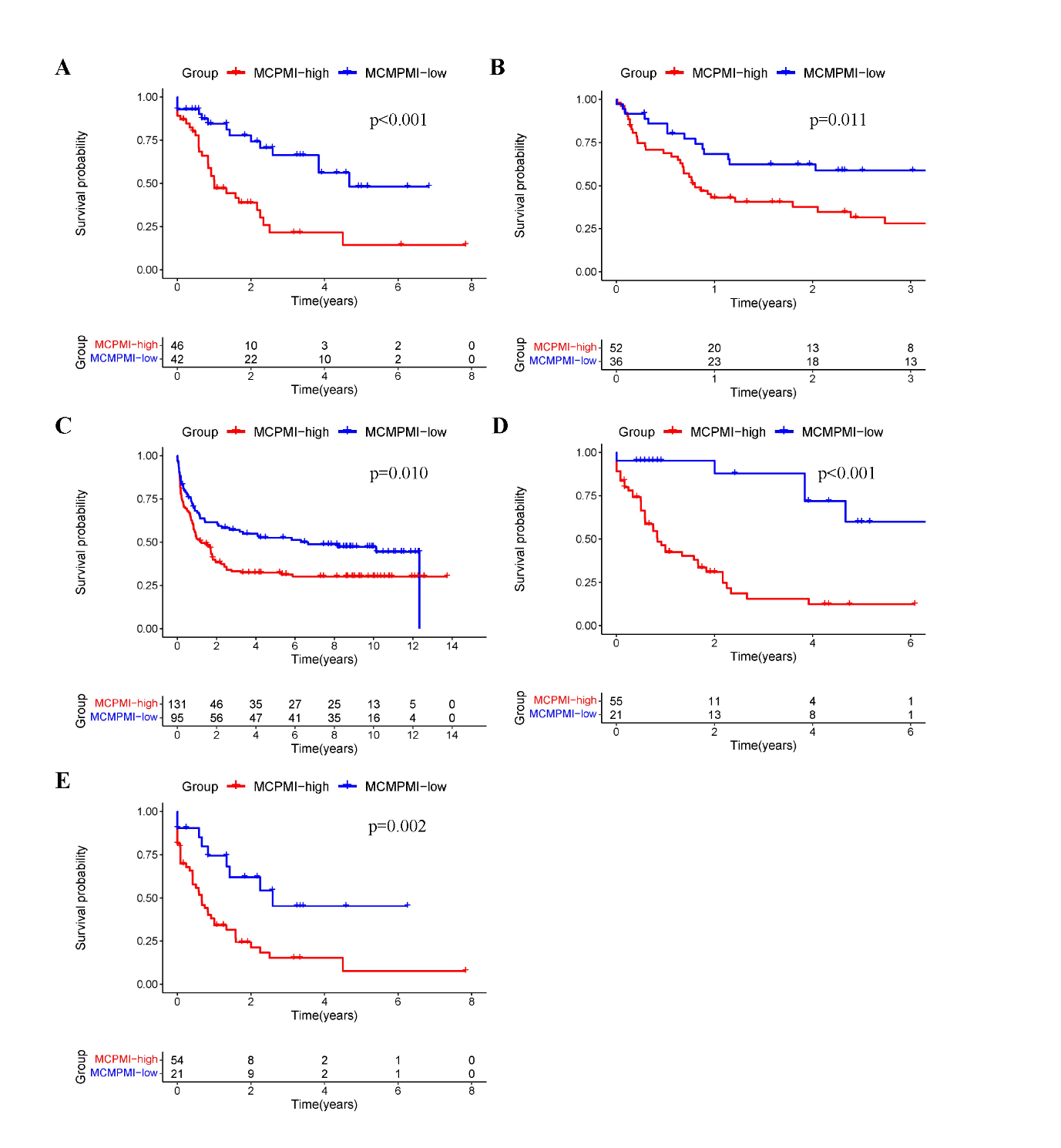
**

**Supplementary Figure 5.** Stratification analysis of MCPMI for its prognostic value. (A-C) Kaplan-Meier survival curves of overall survival for AML patients younger than 60 years based on the MCPMI in the TCGA set (A), GSE12417 set (B) and GSE37642 set (C). (E and F) Kaplan-Meier survival curves of overall survival for AML patients with WBC count＜19^9/L (E) and WBC count≥19^9/L (F) based on the MCPMI in the TCGA set.

**
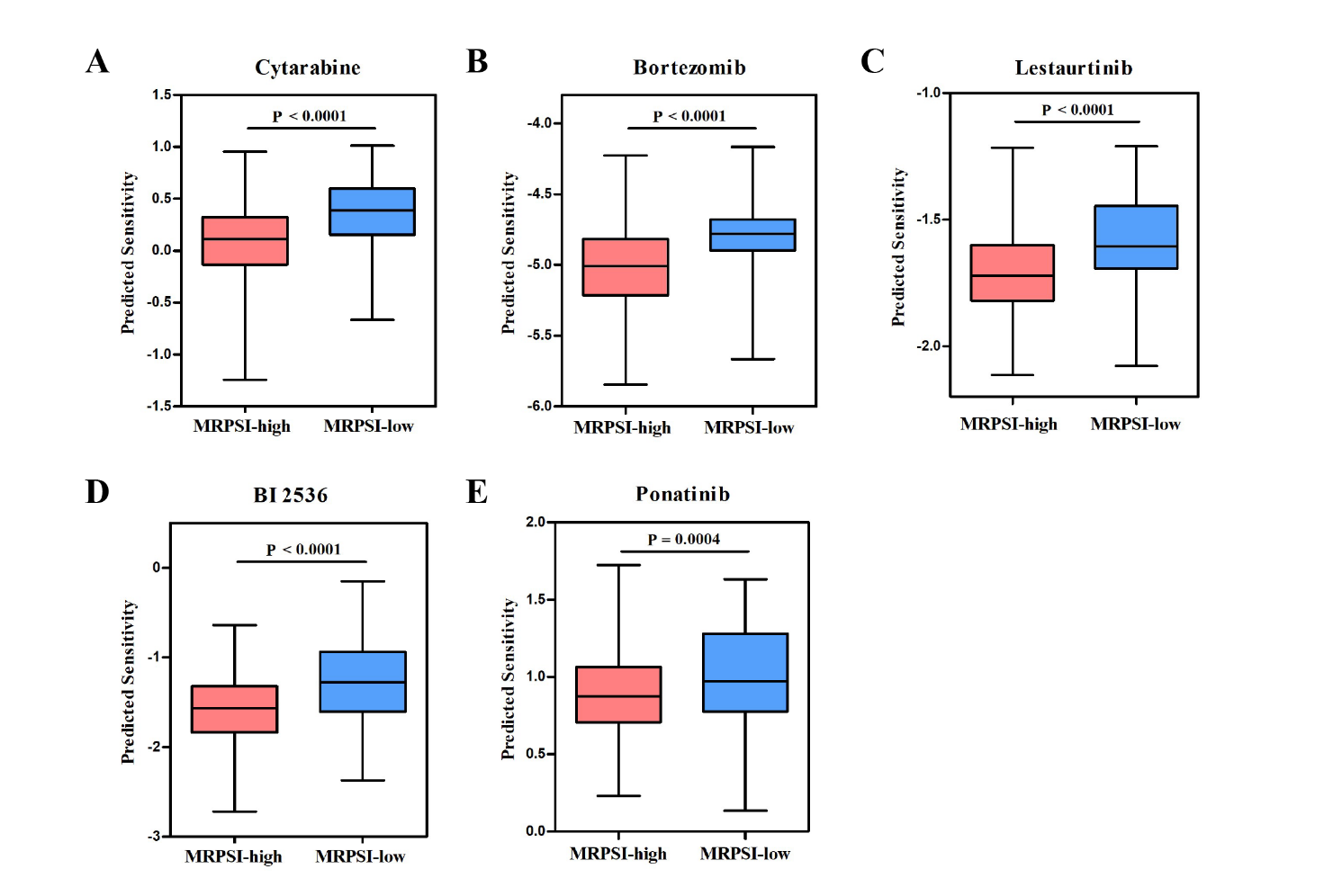
**

**Supplementary Figure 6.** Relationships between MRPSI and drug response in AML patients. (A-E) Boxplots evaluating responses to the chemotherapeutics cytarabine (A), bortezomib (B), lestaurtinib (C), BI 2536 (D) and ponatinib (E) between MRPSI-high and MRPSI-low patients.

## Supplementary Tables

**Supplementary Table 1.** Demographic characteristics of AML patients in three cohorts.

| Characteristics | TCGA | GSE12417 | GSE37642 |
| --- | --- | --- | --- |
| Case No.  Age, n (%) | 151 | 162 | 417 |
| ＜60 | 88 (58.3) | 132 (81.5) | 226 (54.2) |
| ≥60 | 63 (41.7) | 30 (18.5) | 191 (45.8) |
| Gender, n (%) |  |  |  |
| Male | 83 (55.0) | - | - |
| Female | 68 (45.0) | - | - |
| FAB, n (%) |  |  |  |
| M0 | 15 (10.0) | 5 (3.0) | 14 (3.3) |
| M1 | 35 (23.2） | 45 (27.8） | 84 (20.1) |
| M2 | 38 (25.2) | 45 (27.8） | 117 (28.1) |
| M3 | 15 (10.0） | - | 19 (4.6) |
| M4 | 29 (19.2） | 42 (26.0） | 104 (24.9) |
| M5 | 15 (10.0) | 19 (11.7） | 47 (11.3) |
| M6 | 2 (1.2) | 6 (3.7） | 15 (3.6) |
| M7 | 1 (0.6) | - | 2 (0.5) |
| Unknown | 1 (0.6) | - | 15 (3.6) |
| FLT3 mutation, n (%) |  |  |  |
| Wild type | 108 (71.5) | - | - |
| Mutant type | 43 (28.5) | - | - |
| IDH mutation, n (%) |  |  |  |
| Wild type | 122 (80.8) | - | - |
| Mutant type | 29 (19.2) | - | - |

**Supplementary Table 2.** Model information about MRPSI.

| MRG1 | Full name | MRG2 | Full name | Coefficient |
| --- | --- | --- | --- | --- |
| FADS1 | Fatty acid desaturase 1 | NEU1 | Neuraminidase 1 | 0.489 |
| SLC2A5 | Solute carrier family 2 member 5 | TBXAS1 | Thromboxane A synthase 1 | 0.594 |
| FADS1 | Fatty acid desaturase 1 | PDE4B | Phosphodiesterase 4B | 0.427 |

**Supplementary Table 3.** The distribution of patients in MRPSI-high and MRPSI-low groups concerning clinical factors.

| Characteristics | TCGA | | |  | GSE12417 | | | |  | GSE37642 | | |
| --- | --- | --- | --- | --- | --- | --- | --- | --- | --- | --- | --- | --- |
|  | MRPSI-high | MRPSI-low | P value |  | MRPSI-high | MRPSI-low | | P value |  | MRPSI-high | MRPSI-low | P value |
| Case No. | 90 | 61 |  |  | 113 | 49 | |  |  | 303 | 114 |  |
| Age, n (%) |  |  | 0.737 |  |  |  | | 0.195 |  |  |  | 0.008 |
| ＜60 | 51 (56.7) | 37 (60.7) |  |  | 89 (78.8) | 43 (87.8) | |  |  | 152 (50.2) | 74 (64.9) |  |
| ≥60 | 39 (43.3) | 24 (39.3) |  |  | 24 (21.2) | 6 (12.2) | |  |  | 151 (49.8) | 40 (35.1) |  |
| Gender, n (%) |  |  | 0.032 |  |  |  | |  |  |  |  |  |
| Male | 56 (62.2) | 27 (44.3) |  |  | - | - | |  |  | - | - |  |
| Female | 34 (37.8) | 34 (55.7) |  |  | - | - | |  |  | - | - |  |
| FAB, n (%) |  |  | <0.001 |  |  |  | | <0.001 |  |  |  | <0.001 |
| M0 | 15 (16.7) | 0 (0) |  |  | 3 (2.7) | 2 (4.1) | |  |  | 12 (4.0) | 2 (1.7) |  |
| M1 | 23 (25.6） | 12 (19.7） |  |  | 40 (35.4） | 5 (10.2） | |  |  | 69 (22.8） | 15 (13.2） |  |
| M2 | 25 (27.8) | 13 (21.3) |  |  | 35 (31.0) | 10 (20.4) | |  |  | 94 (31.0) | 23 (20.2) |  |
| M3 | 3 (3.3） | 12 (19.7） |  |  | - | - | |  |  | 6 (2.0） | 13 (11.4） |  |
| M4 | 12 (13.3） | 17 (27.8） |  |  | 26 (23.0） | 16 (32.6） | |  |  | 70 (23.1） | 34 (29.9） |  |
| M5 | 8 (8.9) | 7 (11.5) |  |  | 5 (4.4) | 14 (28.6) | |  |  | 24 (7.9) | 23 (20.2) |  |
| M6 | 2 (2.2) | 0 (0) |  |  | 4 (3.5) | 2 (4.1) | |  |  | 13 (4.3) | 2 (1.7) |  |
| M7 | 1 (1.1) | 0 (0) |  |  | - | - | |  |  | 2 (0.6) | 0 (0) |  |
| Unknown | 1 (1.1) | 0 (0) |  |  | - | - | |  |  | 13 (4.3) | 2 (1.7) |  |
| FLT3 mutation, n (%) |  |  | 0.271 |  |  |  | |  |  |  |  |  |
| Wild type | 61 (67.8) | 47 (77.0) |  |  | - | - | |  |  | - | - |  |
| Mutant type | 29 (32.2) | 14 (23.0) |  |  | - | - | |  |  | - | - |  |
| IDH mutation, n (%) |  |  | 0.835 |  |  |  | |  |  |  |  |  |
| Wild type | 72 (80.0) | 50 (82.0) |  |  | - | - | |  |  | - | - |  |
| Mutant type | 18 (20.0) | 11 (18.0) |  |  | - | - | |  |  | - | - |  |
| Blast cell, n (%) |  |  | 0.868 |  |  |  | |  |  |  |  |  |
| ≥71% | 48 (53.3) | 31 (50.8) |  |  | - | - | |  |  | - | - |  |
| ＜71% | 42 (46.7) | 30 (49.2) |  |  | - | - | |  |  | - | - |  |
| WBC count, n (%) |  |  | 0.621 |  |  |  | |  |  |  |  |  |
| ≥19^9/L | 43 (47.8) | 32 (52.5) |  |  | - | - | |  |  | - | - |  |
| ＜19^9/L | 47 (52.2) | 29 (47.5) |  |  | - | - |  | |  | - | - |  |

| Characteristics | TCGA | | | |  | GSE12417 | | | |  | GSE37642 | | | |
| --- | --- | --- | --- | --- | --- | --- | --- | --- | --- | --- | --- | --- | --- | --- |
|  | MCPMI-high | MCPMI-low | | P value |  | MCPMI-high | MCPMI-low | | P value |  | MCPMI-high | MCPMI-low | | P value |
| Case No. | 109 | 42 | |  |  | 126 | 36 | |  |  | 322 | 95 | |  |
| Age, n (%) |  |  | | <0.001 |  |  |  | | <0.001 |  |  |  | | <0.001 |
| ＜60 | 46 (42.2) | 42 (100) | |  |  | 96 (76.2) | 36 (100) | |  |  | 131 (40.7) | 95 (100) | |  |
| ≥60 | 63 (57.8) | 0 (0) | |  |  | 30 (23.8) | 0 (0) | |  |  | 191 (59.3) | 0 (0) | |  |
| Gender, n (%) |  |  | | 0.070 |  |  |  | |  |  |  |  | |  |
| Male | 65 (59.6) | 18 (42.9) | |  |  | - | - | |  |  | - | - | |  |
| Female | 44 (40.4) | 24 (57.1) | |  |  | - | - | |  |  | - | - | |  |
| FAB, n (%) |  |  | | 0.089 |  |  |  | | 0.001 |  |  |  | | <0.001 |
| M0 | 14 (12.9) | 1 (2.4) | |  |  | 3 (2.4) | 2 (5.6) | |  |  | 12 (3.7) | 2 (2.1) | |  |
| M1 | 27 (24.8） | 8 (19.0） | |  |  | 42 (33.3） | 3 (8.3） | |  |  | 71 (22.0） | 13 (13.7） | |  |
| M2 | 28 (25.7) | 10 (23.8) | |  |  | 39 (31.0) | 6 (16.7) | |  |  | 100 (31.0) | 17 (17.9) | |  |
| M3 | 6 (5.5） | 9 (21.5） | |  |  | - | - | |  |  | 10 (3.1） | 9 (9.5） | |  |
| M4 | 19 (17.4） | 10 (23.8） | |  |  | 29 (23.0） | 13 (36.1） | |  |  | 72 (22.4） | 32 (33.7） | |  |
| M5 | 11 (10.1) | 4 (9.5) | |  |  | 9 (7.1) | 10 (27.8) | |  |  | 28 (8.7) | 19 (20.0) | |  |
| M6 | 2 (1.8) | 0 (0) | |  |  | 4 (3.2) | 2 (5.6) | |  |  | 13 (4.0) | 2 (2.1) | |  |
| M7 | 1 (0.9) | 0 (0) | |  |  | - | - | |  |  | 1 (0.3) | 1 (1.1) | |  |
| Unknown | 1 (0.9) | 0 (0) | |  |  | - | - | |  |  | 15 (4.8) | 0 (0) | |  |
| FLT3 mutation, n (%) |  |  | | 0.158 |  |  |  | |  |  |  |  | |  |
| Wild type | 74 (67.9) | 34 (81.0) | |  |  | - | - | |  |  | - | - | |  |
| Mutant type | 35 (32.1) | 8 (19.0) | |  |  | - | - | |  |  | - | - | |  |
| IDH mutation, n (%) |  |  | | 0.068 |  |  |  | |  |  |  |  | |  |
| Wild type | 84 (77.1) | 38 (90.5) | |  |  | - | - | |  |  | - | - | |  |
| Mutant type | 25 (22.9) | 4 (9.5) | |  |  | - | - | |  |  | - | - | |  |
| Blast cell, n (%) |  |  | | 0.203 |  |  |  | |  |  |  |  | |  |
| ≥71% | 61 (56.0) | 18 (42.9) | |  |  | - | - | |  |  | - | - | |  |
| ＜71% | 48 (44.0) | 24 (57.1) | |  |  | - | - | |  |  | - | - | |  |
| WBC count, n (%) |  |  | | 1.000 |  |  |  | |  |  |  |  | |  |
| ≥19^9/L | 54 (49.5) | 21 (50.0) | |  |  | - | - | |  |  | - | - |  | |
| ＜19^9/L | 55 (50.5) | | 21 (50.0) |  |  | - | - |  | |  | - | - |  | |

**Supplementary Table 4.** The distribution of patients in MCPMI-high and MCPMI-low groups concerning clinical factors.
